# Functional Characterization of NAC and MYB Transcription Factors Involved in Regulation of Biomass Production in Switchgrass (*Panicum virgatum*)

**DOI:** 10.1371/journal.pone.0134611

**Published:** 2015-08-06

**Authors:** Ruiqin Zhong, Youxi Yuan, John J. Spiekerman, Joshua T. Guley, Janefrances C. Egbosiuba, Zheng-Hua Ye

**Affiliations:** Department of Plant Biology, University of Georgia, Athens, Georgia, 30602, United States of America; Iowa State University, UNITED STATES

## Abstract

Switchgrass is a promising biofuel feedstock due to its high biomass production and low agronomic input requirements. Because the bulk of switchgrass biomass used for biofuel production is lignocellulosic secondary walls, studies on secondary wall biosynthesis and its transcriptional regulation are imperative for designing strategies for genetic improvement of biomass production in switchgrass. Here, we report the identification and functional characterization of a group of switchgrass transcription factors, including several NACs (PvSWNs) and a MYB (PvMYB46A), for their involvement in regulating secondary wall biosynthesis. *PvSWNs* and *PvMYB46A* were found to be highly expressed in stems and their expression was closely associated with sclerenchyma cells. Overexpression of PvSWNs and PvMYB46A in Arabidopsis was shown to result in activation of the biosynthetic genes for cellulose, xylan and lignin and ectopic deposition of secondary walls in normally parenchymatous cells. Transactivation and complementation studies demonstrated that PvSWNs were able to activate the SNBE-driven GUS reporter gene and effectively rescue the secondary wall defects in the Arabidopsis *snd1 nst1* double mutant, indicating that they are functional orthologs of Arabidopsis SWNs. Furthermore, we showed that PvMYB46A could activate the SMRE-driven GUS reporter gene and complement the Arabidopsis *myb46 myb83* double mutant, suggesting that it is a functional ortholog of Arabidopsis MYB46/MYB83. Together, these results indicate that PvSWNs and PvMYB46A are transcriptional switches involved in regulating secondary wall biosynthesis, which provides molecular tools for genetic manipulation of biomass production in switchgrass.

## Introduction

Switchgrass (*Panicum virgatum* L.) is considered to be one of the leading renewable and sustainable feedstock crops for the production of biofuels. It is a perennial grass species with a high yield of aboveground biomass and high productivity when grown on marginal land, which is in part due to its beneficial physiological traits such as the production of deep roots and the utilization of C_4_ photosynthetic metabolism [1,2]. Furthermore, when compared with first-generation biofuel crops, switchgrass has been shown to be extremely efficient in its consumption of water and nutrients with low fertilizer inputs during development [1,2]. Because the switchgrass biomass used for biofuel production is mainly the lignocellulosic cell wall residues from stems and leaves, much attention has been paid toward altering cell wall composition in order to reduce biomass recalcitrance for conversion into biofuels.

Several lignin biosynthetic genes have been targeted for reduction in lignin content in switchgrass as lignin is one of the main factors contributing to biomass recalcitrance. Downregulation of *Caffeic Acid O-Methyltransferase* (*COMT*) in switchgrass by RNA interference (RNAi) resulted in a reduction in lignin content and consequently, a higher yield of biomass conversion into ethanol compared with the wild type under the same saccharification and fermentation conditions [3]. Similarly, downregulation of *Cinnamyl Alcohol Dehydrogenase* (*CAD*) or *4-Coumarate*:*Coenzyme A Ligase* (*4CL*) in switchgrass by RNAi caused a reduction in lignin content and an increase in biomass saccharification efficiency [4,5]. A reduction in lignin content and an improvement in saccharification efficiency in switchgrass were also achieved by overexpression of a MYB transcription factor, PvMYB4, which represses the expression of lignin biosynthetic genes [6]. These studies provide convincing evidence that a reduction in lignin content in switchgrass is a feasible approach to overcome biomass recalcitrance.

To develop switchgrass as a bioenergy feedstock, it is important to unravel the mechanisms underlying the biomass biosynthesis and the transcriptional regulation of biomass production. Uncovering genes involved in biomass biosynthesis and its regulation may provide additional tools to further manipulate switchgrass biomass yield and composition tailored for biofuel production. Genes involved in lignin biosynthesis in switchgrass have been identified using an inducible switchgrass cell suspension system and several of them have been confirmed for their involvement in lignin biosynthesis by RNAi downregulation of their expression in switchgrass [7]. Four *Sucrose Synthase* (*SUS*) genes, which are potentially involved in the supply of UDP-glucose for cellulose biosynthesis, have been functionally characterized in switchgrass. Overexpression of one of them, PvSUS1, in switchgrass led to increased plant height and biomass, suggesting the potential usefulness of *SUS* genes as targets to increase biomass production in switchgrass [8]. In addition, as mentioned above, PvMYB4 has been shown to be a transcriptional repressor regulating lignin biosynthesis [6]. Because the bulk of switchgrass biomass is from sclerenchyma cells with heavily thickened secondary walls, further studies of genes involved in secondary wall biosynthesis and its regulation will be important for better utilization of switchgrass biomass.

In this report, we present the identification and functional characterization of several NAC and MYB genes involved in regulation of secondary wall biosynthesis. In Arabidopsis, a group of secondary wall NACs (SWNs) function as master transcriptional switches activating the entire secondary wall biosynthesis program in vessels and fibers [9]. Arabidopsis SWNs are vessel-specific VND1 to VND7 and fiber-specific SND1/NST1/2, and when overexpressed, they are sufficient to activate the biosynthetic pathways for cellulose, xylan and lignin as well as the genes for programmed cell death [10–19]. They regulate their downstream target genes, including a number of transcription factors and secondary wall biosynthetic genes, by binding to the secondary wall NAC binding element (SNBE) consisting of a 19-bp imperfect palindromic consensus sequence, (T/A)NN(C/T)(T/C/G)TNNNNNNNA(A/C)GN(A/C/T)(A/T) [20]. The SWN-regulated downstream transcription factors include secondary wall-associated SND2, SND3, MYB20, MYB42, MYB43, MYB46, MYB52, MYB58, MYB63, MYB69, MYB83, MYB85, MYB103 and KNAT7 [21–24], and among them, MYB46 and MYB83 act as second-level master switches and bind to the secondary wall MYB responsive element [SMRE; ACC(A/T)A(A/C)(T/C)] to activate their target genes [25,26]. Orthologs of SWNs and MYB46/83 from rice, maize and *Brachypodium* have also been demonstrated to regulate secondary wall biosynthesis, indicating the evolutionary conservation of the SWN-mediated transcriptional network controlling secondary wall biosynthesis [27–29]. By searching the switchgrass genome, we have identified 14 SWN homologs (PvSWNs) and two MYB46/MYB83 homologs (PvMYB46A/B). We show that PvSWNs and PvMYB46A are capable of activating the expression of the biosynthetic genes for cellulose, xylan and lignin, leading to ectopic deposition of secondary walls when overexpressed in Arabidopsis. We further demonstrate that PvSWNs can effectively activate the expression of the SNBE-driven GUS reporter gene and complement the Arabidopsis *snd1 nst1* double mutant. Likewise, PvMYB46A is able to activate the SMRE-driven GUS reporter gene and rescue the Arabidopsis *myb46 myb83* double mutant phenotypes. Our findings indicate that PvSWNs and PvMYB46A are functional orthologs of Arabidopsis SWNs and MYB46/MYB83, respectively, and are involved in regulating secondary wall biosynthesis in switchgrass.

## Results

### Expression analysis of PvSWNs

**Fig 1:**
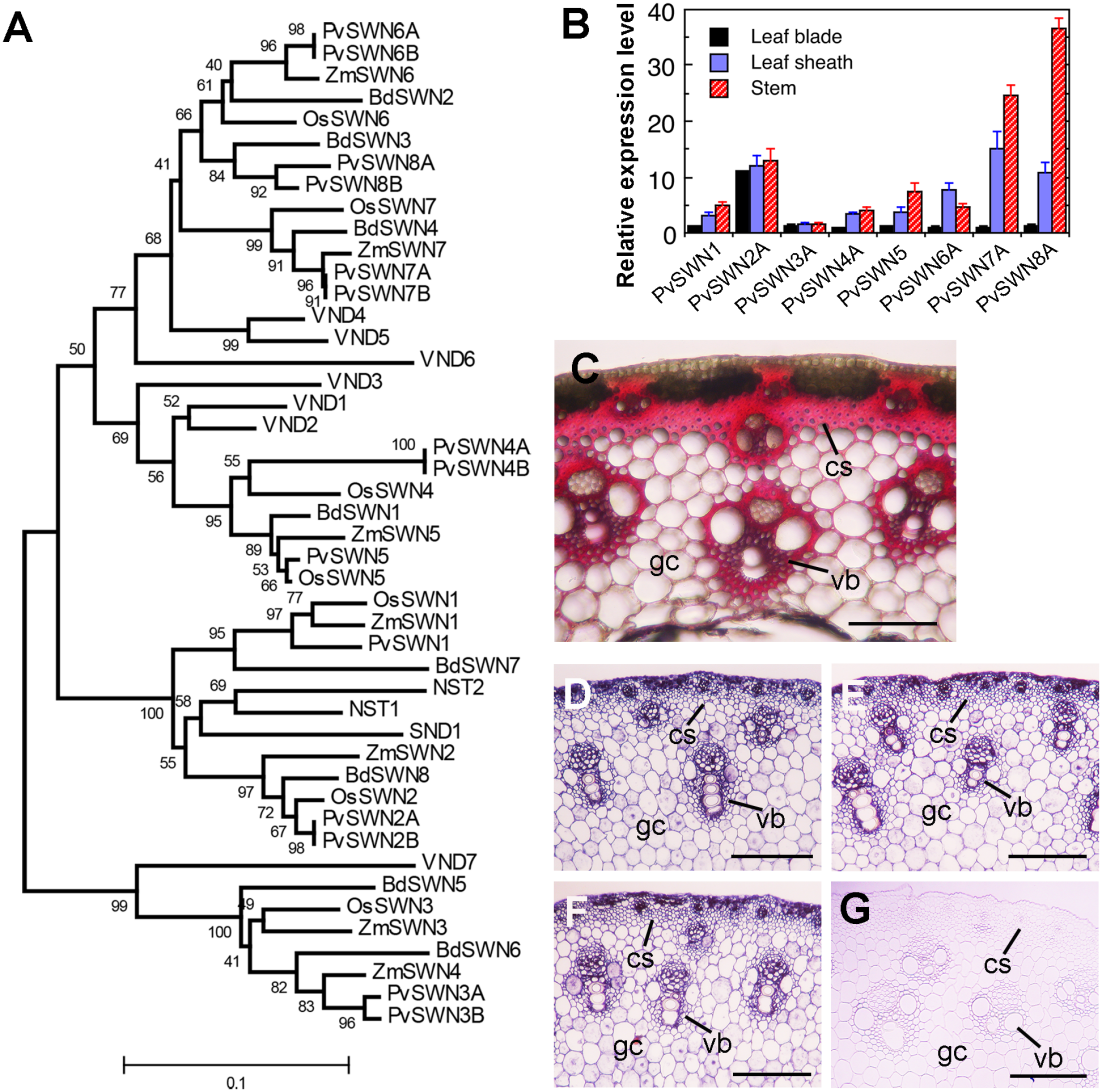
Phylogenetic and expression analyses of PvSWNs. (A) phylogenetic relationship of PvSWNs with their orthologs from Arabidopsis (VNDs/SND1/NSTs), rice (OsSWNs), maize (ZmSWNs) and *Brachypodium* (BdSWNs). The phylogenetic tree was constructed with the neighbor-joining algorithm and the bootstrap values resulted from 1,000 replicates are shown as percentages at the nodes. The 0.1 scale denotes 10% change. (B) Quantitative PCR analysis of the expression of *PvSWNs* in switchgrass organs, including leaf blades, leaf sheaths and stems. The expression level of *PvSWNs* in leaf blades was set to 1. Error bars represent the SE of three biological replicates. (C) Cross section of a switchgrass stem showing lignified cell walls, which were stained red by phloroglucinol-HCl. Bar = 141 μm. (D-G) In situ localization of *PvSWN2A* (D), *PvSWN7A* (E) and *PvSWN8A* (F) mRNAs in switchgrass stems. Cross sections of stems were hybridized with digoxigenin-labeled antisense *PvSWN* RNA probes (D-F) or sense *PvSWN2A* RNA probe (G) and the hybridization signals, which were detected by alkaline phosphatase-conjugated antibodies, were shown in purple. Note that the majority of cells showing the hybridization signals were matched with those having lignified secondary walls as seen in (C) although those patches of non-lignified cortical cells below the epidermis also showed the hybridization signals, which could be due to non-specific hybridization. Bars = 153 μm. cs, cortical sclerenchyma; gc, ground cell; vb, vascular bundle.

In the switchgrass (*Panicum virgatum*) genome, there exist 14 NAC genes that are close homologs of Arabidopsis secondary wall NAC master switches (SWNs). They appear to be divided into three subgroups based on the phylogenetic analysis (Fig 1A). PvSWN1/2A/2B are grouped closely with SND1/NST1/2, PvSWN3A/3B are grouped closely with VND7, and PvSWN4-8 are grouped closely with VND1-6. For each PvSWN, there exist close homologs in rice, maize and/or *Brachypodium* (Fig 1A). Except PvSWN1 and PvSWN5, the rest of PvSWNs form pairs, of which the two members share at least 92.4% similarity at the amino acid level. Since switchgrass is a tetraploid species, the gene pairing of PvSWNs is likely due to the increased ploidy level. In this study, eight PvSWNs, including PvSWN1, PvSWN5 and one member from each pair of other PvSWNs, were functionally characterized. Quantitative PCR analysis showed that *PvSWN* genes were all expressed in leaf blades, leaf sheaths and stems, and among them, *PvSWN2A*, *PvSWN7A* and *PvSWN8A* exhibited a high level expression in stems (Fig 1B). Examination of lignin deposition in mature switchgrass stems showed that in addition to its heavy deposition in the secondary walls in xylem and cortical sclerenchyma cells, lignin was present in the walls of ground cells (Fig 1C). A low level of lignin staining was also observed in the walls of epidermal cells and some phloem parenchyma cells. In situ hybridization revealed that *PvSWN2A*, *PvSWN7A* and *PvSWN8A* were expressed in xylem bundle cells, cortical sclerenchyma cells, and ground cells in switchgrass stems (Fig 1D–1F), which were matched with the lignin-staining pattern in these tissues (Fig 1C). However, hybridization signals were also seen in those patches of cortical cells below epidermis that did not deposit lignified secondary walls, which could be resulted from non-specific hybridization. The control stem section hybridized with the sense probe of *PvSWN2A* exhibited no hybridization signal (Fig 1G). These findings indicate that the expression of *PvSWN2A*, *PvSWN7A* and *PvSWN8A* is associated with secondary wall deposition and lignification in switchgrass stems.

### Overexpression of PvSWNs in Arabidopsis results in ectopic deposition of cellulose, xylan and lignin

**Fig 2:**
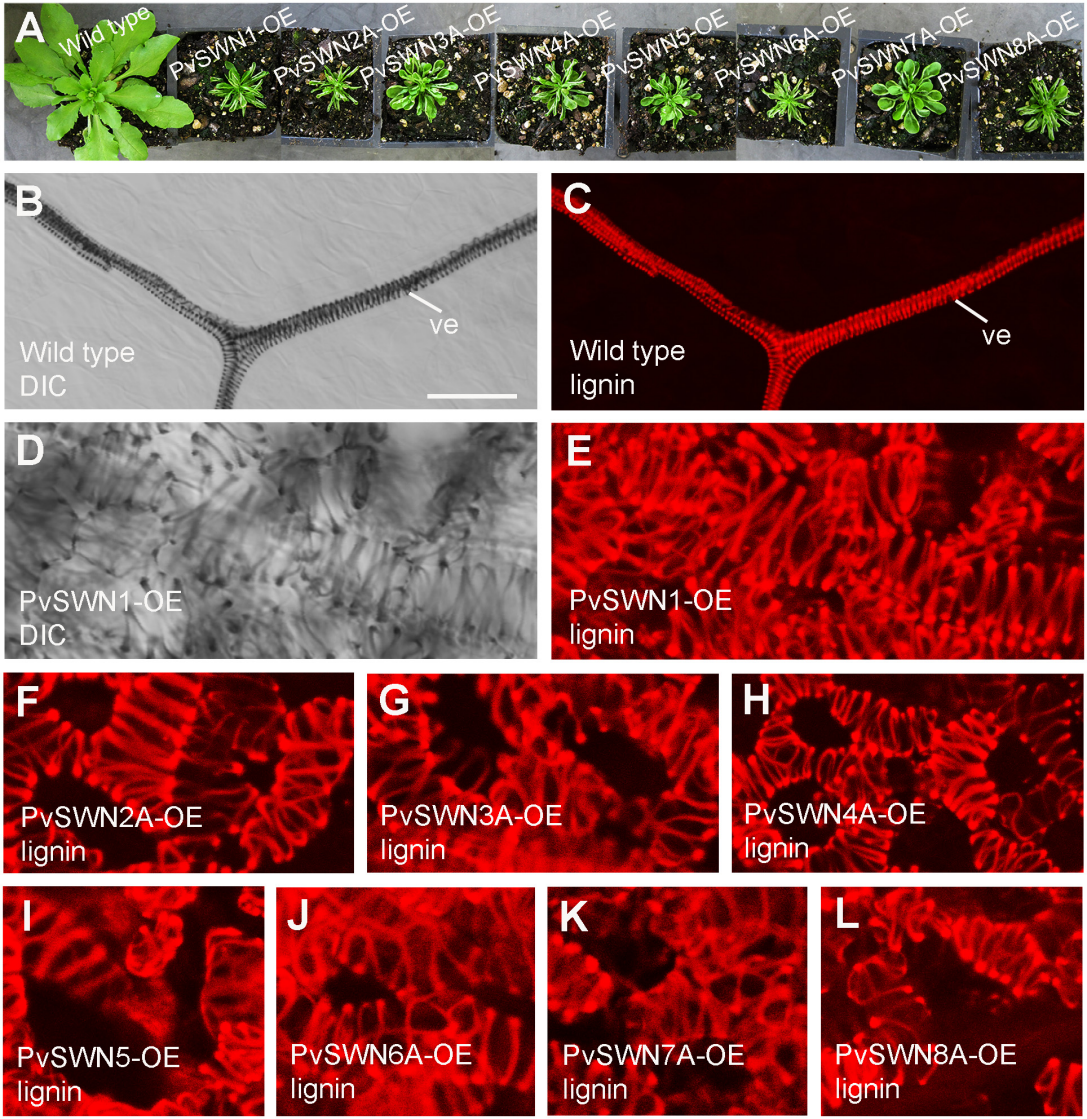
Ectopic deposition of lignified secondary walls in Arabidopsis leaves overexpressing PvSWNs. Three-week-old wild-type and transgenic Arabidopsis seedlings were used for phenotypic analysis. (A) PvSWN overexpressors (PvSWN-OE) showing their smaller rosette sizes and curly leaves compared with the wild type. (B, C) Differential interference contrast (DIC) image (B) and lignin autofluorescence image (C) of a wild-type leaf showing helical secondary wall thickening in veins. (D, E) DIC (D) and lignin autofluorescence image (E) of a PvSWN1-OE leaf showing thick lignified walls with a reticulated pattern in mesophyll cells. (F to L) Lignin autofluorescence images showing ectopic deposition of reticulated, lignified secondary walls in leaves of PvSWN2A-OE (F), PvSWN3A-OE (G), PvSWN4A-OE (H), PvSWN5-OE (I), PvSWN6A-OE (J), PvSWN7A-OE (K) and PvSWN8A-OE (L). ve, vein. Bar in (B) = 28 μm for (B to L).

To find out whether PvSWNs are involved in regulating secondary wall biosynthesis, we investigated the effects of PvSWN overexpression on secondary wall deposition in transgenic Arabidopsis. The full-length cDNAs of *PvSWNs* driven by the *Cauliflower mosaic virus* (CaMV) 35S promoter were introduced into wild-type Arabidopsis and at least 30 transgenic plants for each PvSWN-OE construct were generated. At least one-third of the transgenic plants for each of the eight constructs had small rosettes with leaves curled upward (Fig 1A), a phenotype similar to that observed in the SND1 overexpressors [13]. Examination of PvSWN-OE leaves revealed that in contrast to the wild-type leaves, which only had lignified helical secondary wall thickening in veins (Fig 2B and 2C), the PvSWN overexpressors had lignified secondary walls in many mesophyll cells (Fig 2D–2L), which were deposited in a helical or reticulated pattern. The ectopically deposited secondary walls in the leaf mesophyll cells most likely restricted the normal expansion of leaves, leading to the small, curly leaf phenotype.

**Fig 3:**
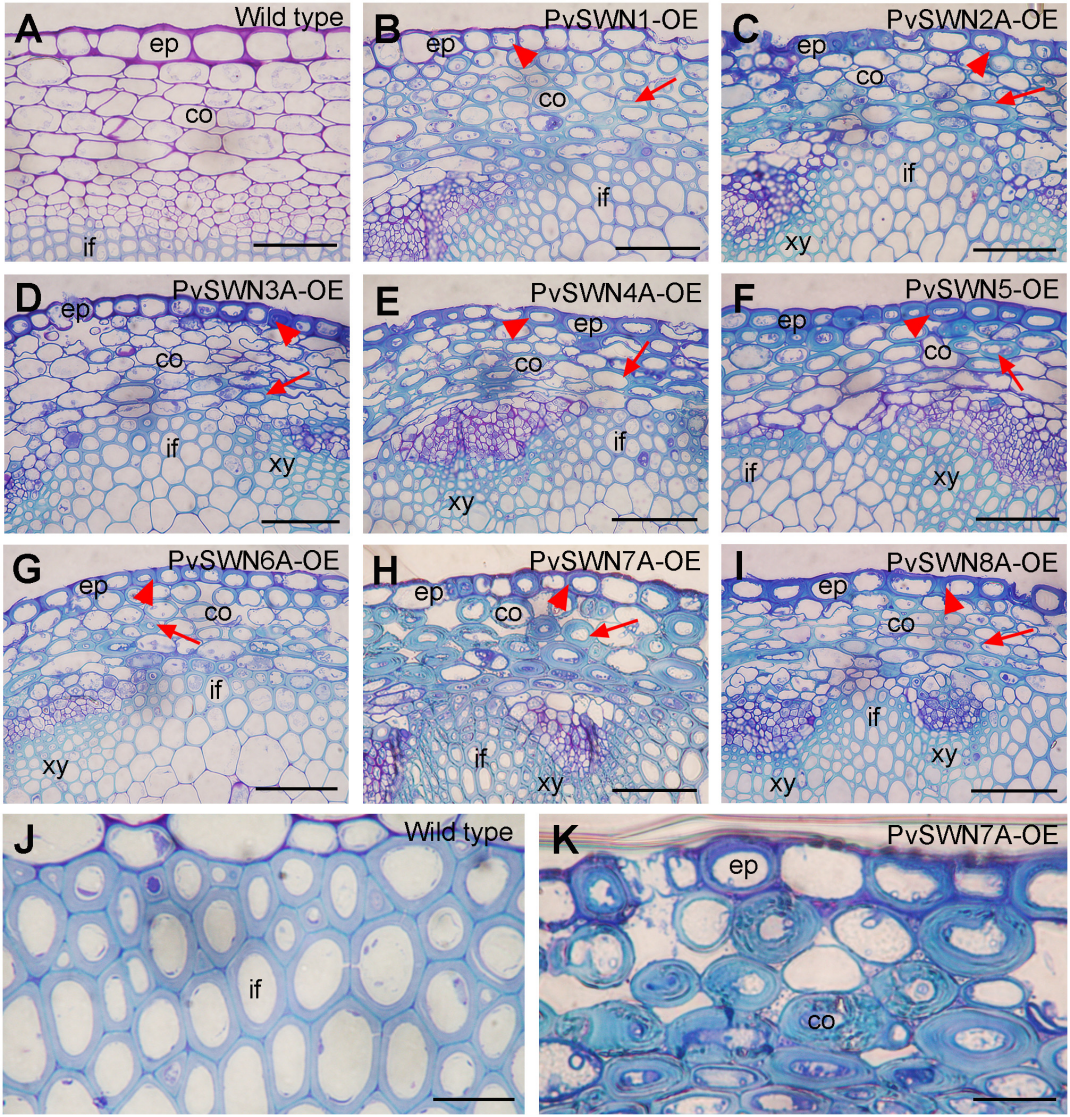
PvSWN overexpression causes ectopic deposition of secondary walls in the epidermal and cortical cells of Arabidopsis stems. Cross sections of stems were stained with toluidine blue and the lignified secondary walls exhibit blue staining. (A) Section of a wild-type stem showing cortical cells with thin primary walls. (B to I) Stem sections of PvSWN overexpressors showing ectopic deposition of secondary walls in the epidermal cells (arrowheads) and cortical cells (arrows). (J, K) An enlarged image of the stem cortical cells of PvSWN7A overexpressor (K) showing much thicker secondary walls than interfascicular fiber cells in the wild type (J). co, cortex; ep, epidermis; if, interfascicular fiber; xy, xylem. Bars = 52 μm for (A to I) and 78 μm for (J, K).

**Fig 4:**
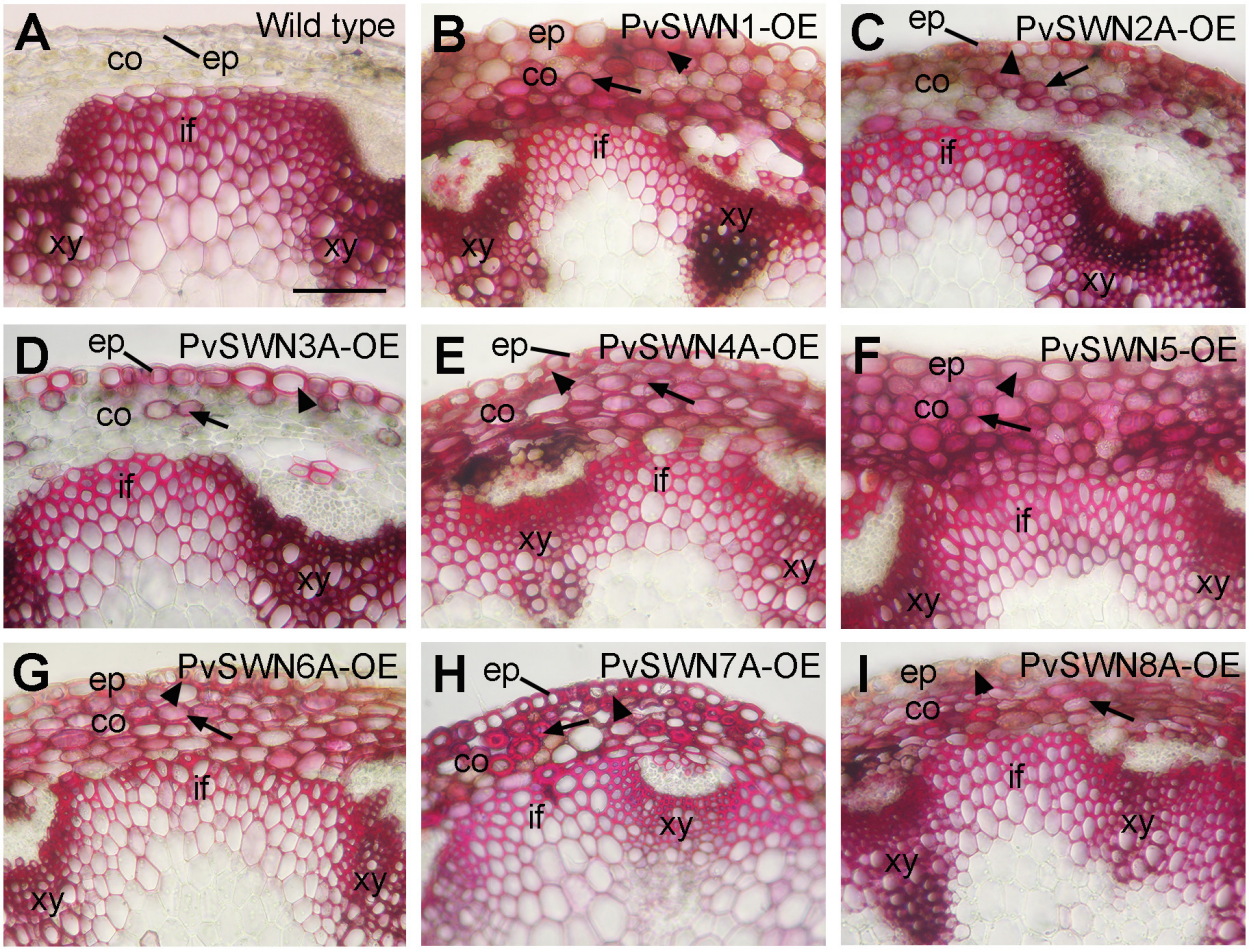
Ectopic deposition of lignin in the epidermal and cortical cells in the stems of PvSWN overexpressors. Cross sections of stems were stained for lignin with phloroglucionol-HCl and the lignified walls are stained red. (A) Section of a wild-type stem showing lignin staining in the walls of interfascicular fibers and xylem cells and its absence in the epidermal and cortical cells. (B to I) Stem sections of PvSWN overexpressors showing ectopic lignin deposition in the epidermal cells (arrowheads) and cortical cells (arrows) in addition to its normal deposition in the interfascicular fibers and xylem cells. co, cortex; ep, epidermis; if, interfascicular fiber; xy, xylem. Bar in (A) = 170 μm for (A to I).

**Fig 5:**
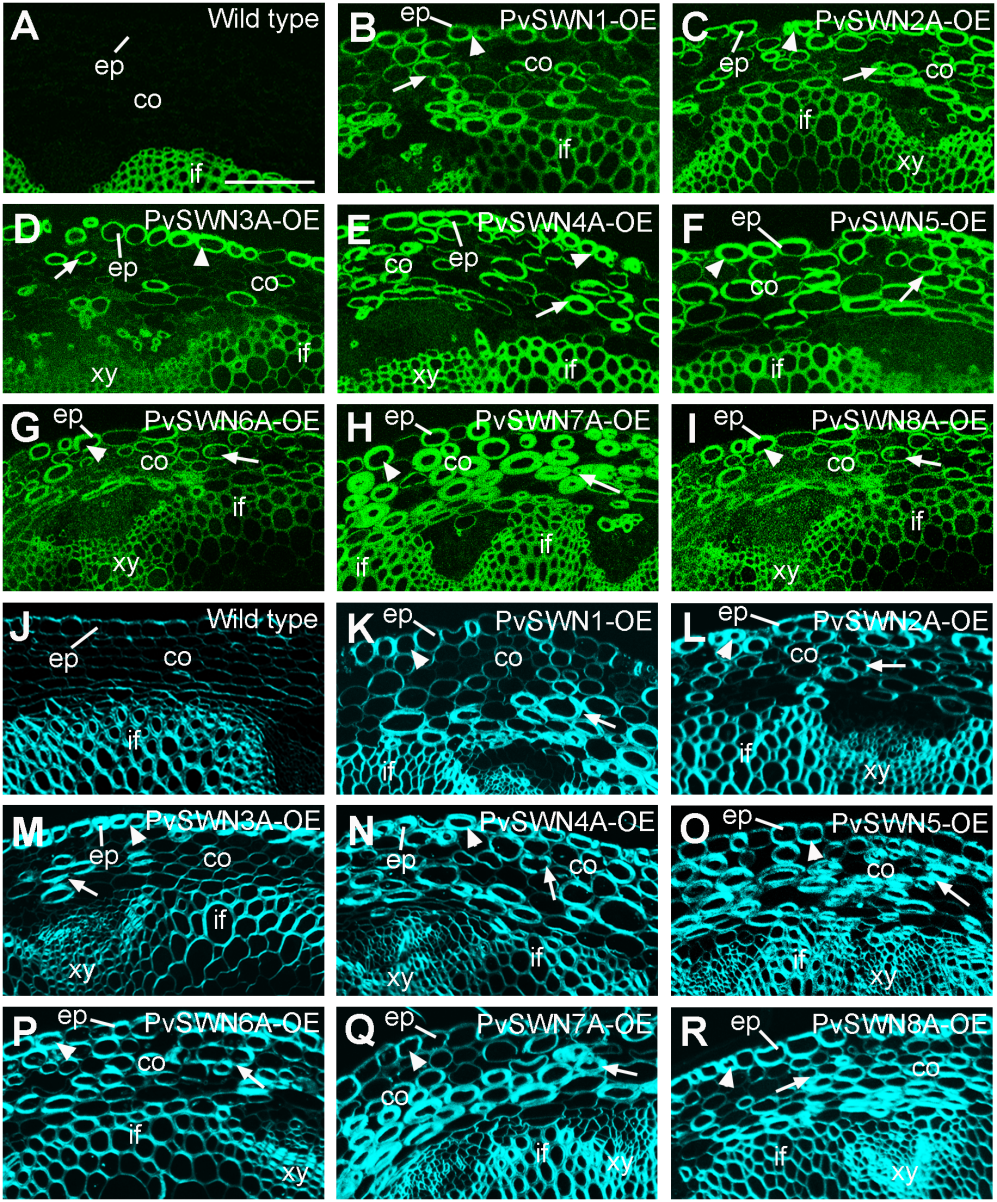
Ectopic deposition of xylan and cellulose in the epidermal and cortical cells in the stems of PvSWN overexpressors. Cross sections of stems were stained for xylan with the xylan-specific monoclonal antibody LM10 (A to I) and for cellulose with Calcofluor White. (A) Section of a wild-type stem showing xylan staining in the walls of interfascicular fibers and its absence in the epidermal and cortical cells. (B to I) Stem sections of PvSWN overexpressors showing ectopic xylan deposition in the epidermal cells (arrowheads) and cortical cells (arrows) in addition to its normal deposition in the interfascicular fibers and xylem cells. (J) Section of a wild-type stem showing the predominant cellulose staining in the walls of interfascicular fibers. (K to R) Stem sections of PvSWN overexpressors showing intense cellulose staining in the epidermal cells (arrowheads) and cortical cells (arrows) in addition to the interfascicular fibers and xylem cells. co, cortex; ep, epidermis; if, interfascicular fiber; xy, xylem. Bar in (A) = 256 μm for (A to R).

The ectopic deposition of secondary walls also occurred in the epidermal and cortical cells of PvSWN-OE stems (Fig 3). The most heavily thickened secondary walls were observed in the epidermal and cortical cells of stems of PvSWN4A-OE (Fig 3E), PvSWN5-OE (Fig 3F) and PvSWN7A-OE (Fig 3H). It was noted that the ectopic secondary walls in some cortical cells of the PvSWN7A-OE stems (Fig 3K) were 2 to 3 times thicker than those of the interfascicular fibers of wild-type stems (Fig 3J). To investigate whether the ectopically deposited cell walls contained all three secondary wall components, stem sections of PvSWN overexpressors were detected for cellulose and lignin by histological staining and for xylan by immunostaining. It was found that although the epidermal and cortical cells of wild-type stems showed no staining for lignin (Fig 4A) and xylan (Fig 5A) and little staining for cellulose (Fig 5J), the ectopically deposited secondary walls in the epidermal and cortical cells of PvSWN-OE stems were heavily stained for lignin (Fig 4B–4I), xylan (Fig 5B–5I) and cellulose (Fig 5K–5R). These results indicate that PvSWN overexpression activates the biosynthetic pathways of cellulose, xylan and lignin, leading to ectopic deposition of secondary walls in normally parenchymatous cells.

### Overexpression of PvSWNs in Arabidopsis induces the expression of secondary wall-associated transcription factors and secondary wall biosynthetic genes

**Fig 6:**
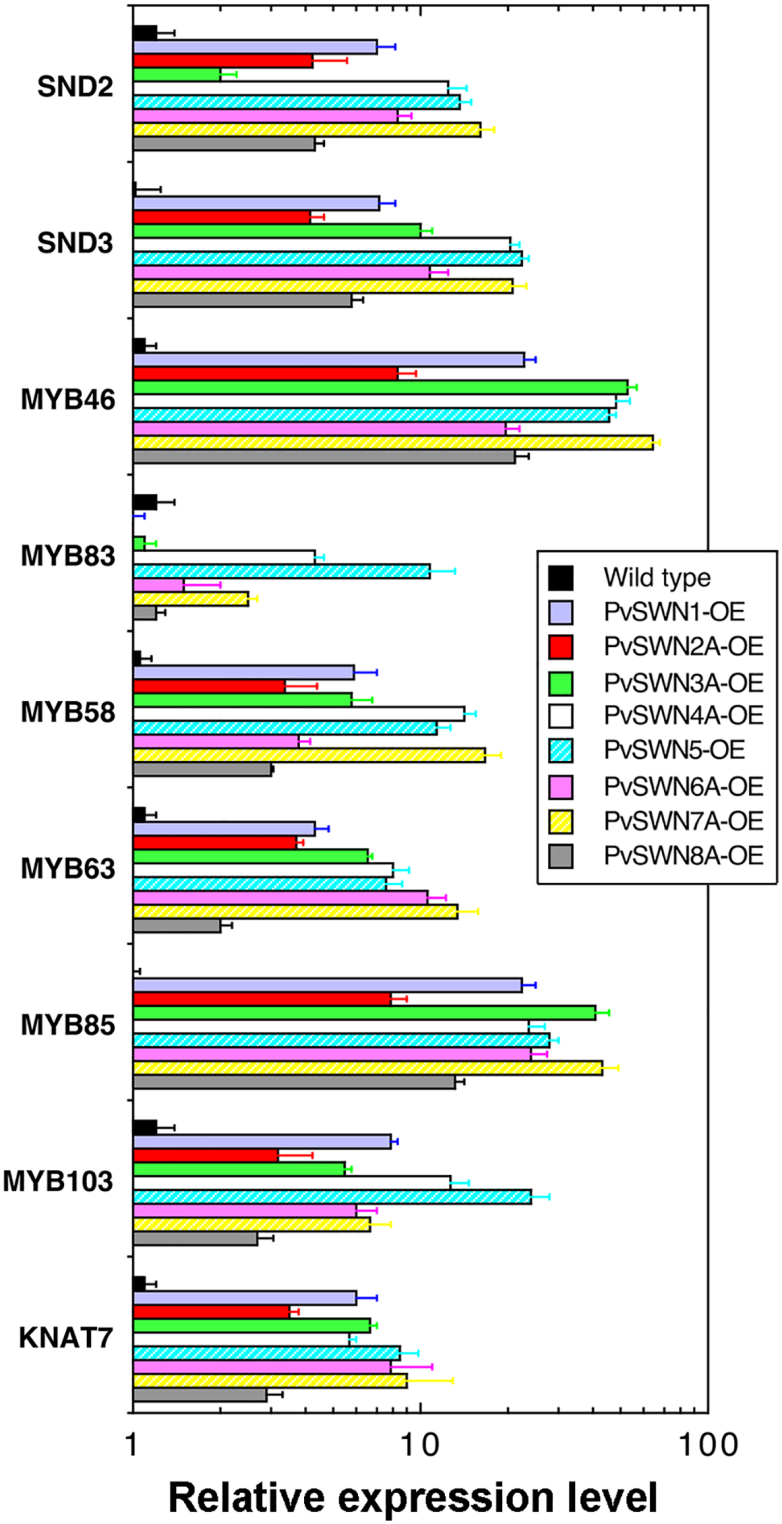
Induction in the expression of secondary wall-associated transcription factors in PvSWN overexpressors. Leaves of 4-week-old transgenic Arabidopsis plants overexpressing PvSWNs were extracted for RNA, which was subsequently used for quantitative PCR analysis of the expression of various transcription factors. The expression level of each gene in the wild type was set to 1. Error bars represent the SE of three biological replicates.

To further substantiate the finding that PvSWNs were capable of activating the secondary wall biosynthetic program, we investigated whether PvSWN overexpression induced the expression of secondary wall-associated transcription factors and secondary wall biosynthetic genes. SND1 has been shown to regulate the expression of a number of downstream transcription factors, including *SND2*, *SND3*, *MYB46*, *MYB83*, *MYB58*, *MYB63*, *MYB85*, *MYB103* and *KNAT7*, all of which have been demonstrated to regulate secondary wall biosynthesis [9]. Quantitative PCR analysis revealed that the expression of all these SND1-regulated transcription factors was highly induced in the PvSWN-OE lines (Fig 6). The induction level of each transcription factor appeared to vary significantly among different PvSWN overexpressors, which may be due to the differential activation strength by PvSWNs.

**Fig 7:**
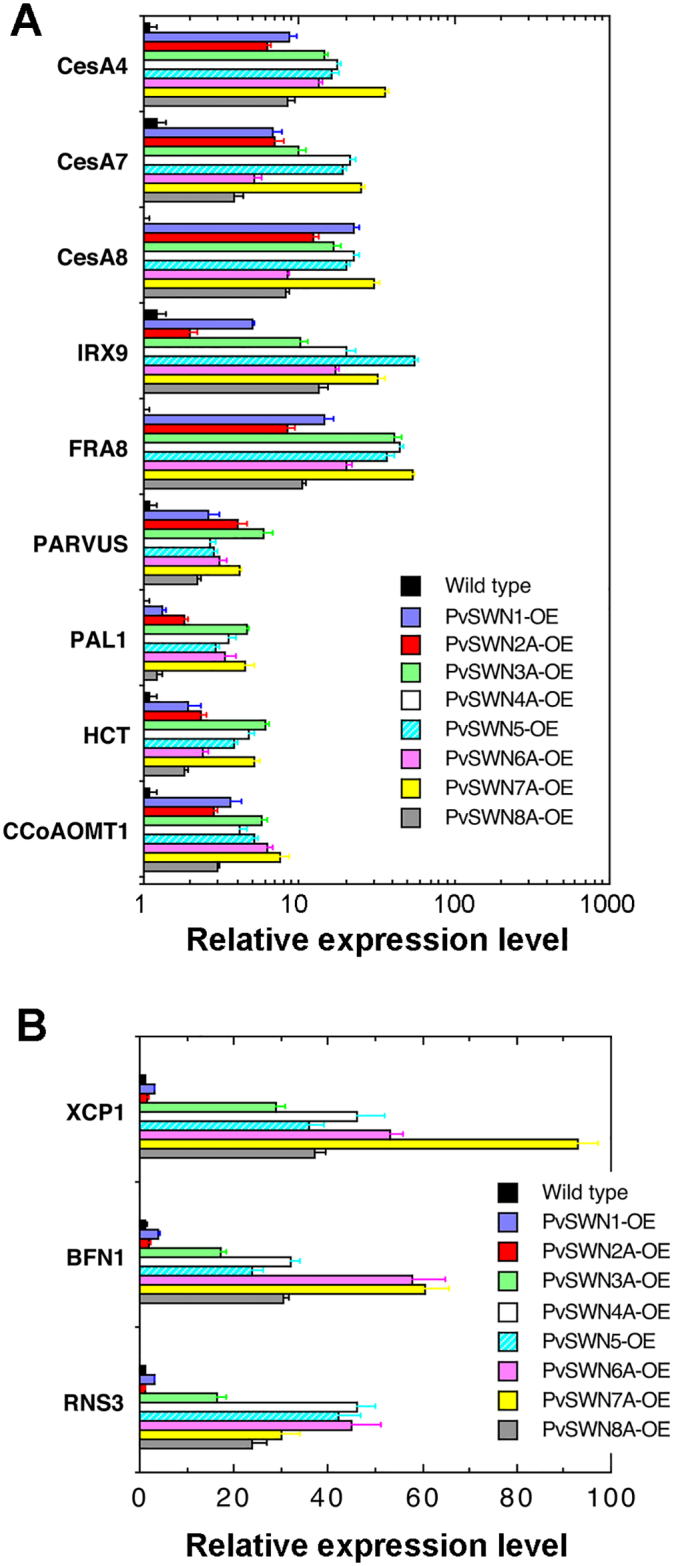
Induction in the expression of secondary wall biosynthetic genes and programmed cell death genes in PvSWN overexpressors. Leaves of 4-week-old transgenic Arabidopsis plants overexpressing PvSWNs were extracted for RNA, which was subsequently used for quantitative PCR analysis of the expression of secondary wall biosynthetic genes (A) and programmed cell death genes (B). The expression level of each gene in the wild type was set to 1. Error bars represent the SE of three biological replicates.

Examination of the expression of secondary wall biosynthetic genes showed that PvSWN overexpression induced the expression of biosynthetic genes for secondary wall cellulose [represented by *Cellulose SynthaseA4* (*CesA4*), *CesA7* and *CesA8*], xylan [represented by *Fragile Fiber8* (*FRA8*) and *Irregular Xylem9* (*IRX9*)], and lignin [represented by *Hydroxycinnamoyl CoA-Shikimate Hydroxycinnamoyl Transferase* (*HCT*) and *Caffeoyl CoA O-Methyltransferase* (*CCoAOMT*)] [30] (Fig 7A). These results demonstrated that congruent with the observed ectopic secondary wall deposition, PvSWN overexpression activated the expression of both secondary wall-associated transcription factors and secondary wall biosynthetic genes.

Arabidopsis vessel-specific SWNs, including VND1 to VND7, have been previously shown to regulate the expression of not only secondary wall biosynthetic genes but also genes involved in programmed cell death [15,17,20]. Quantitative PCR analysis revealed that three representative genes involved in programmed cell death, *Bifunctional Nuclease1* (*BFN1*), *Ribonuclease3* (*RNS3*) and *Xylem Cysteine Peptidase1* (*XCP1*), were highly induced by overexpression of PvSWN3A, PvSWN4A, PvSWN5, PvSWN6A, PvSWN7A and PvSWN8A (Fig 7B), which are close homologs of VND1 to VND7 (Fig 1A). In contrast, overexpression of PvSWN1 and PvSWN2A, which are close homologs of SND1 and NST1/2, did not significantly affect the expression levels of *BFN1*, *RNS3* and *XCP1* (Fig 7B).

### Complementation of the Arabidopsis *snd1 nst1* mutant by PvSWNs

**Fig 8:**
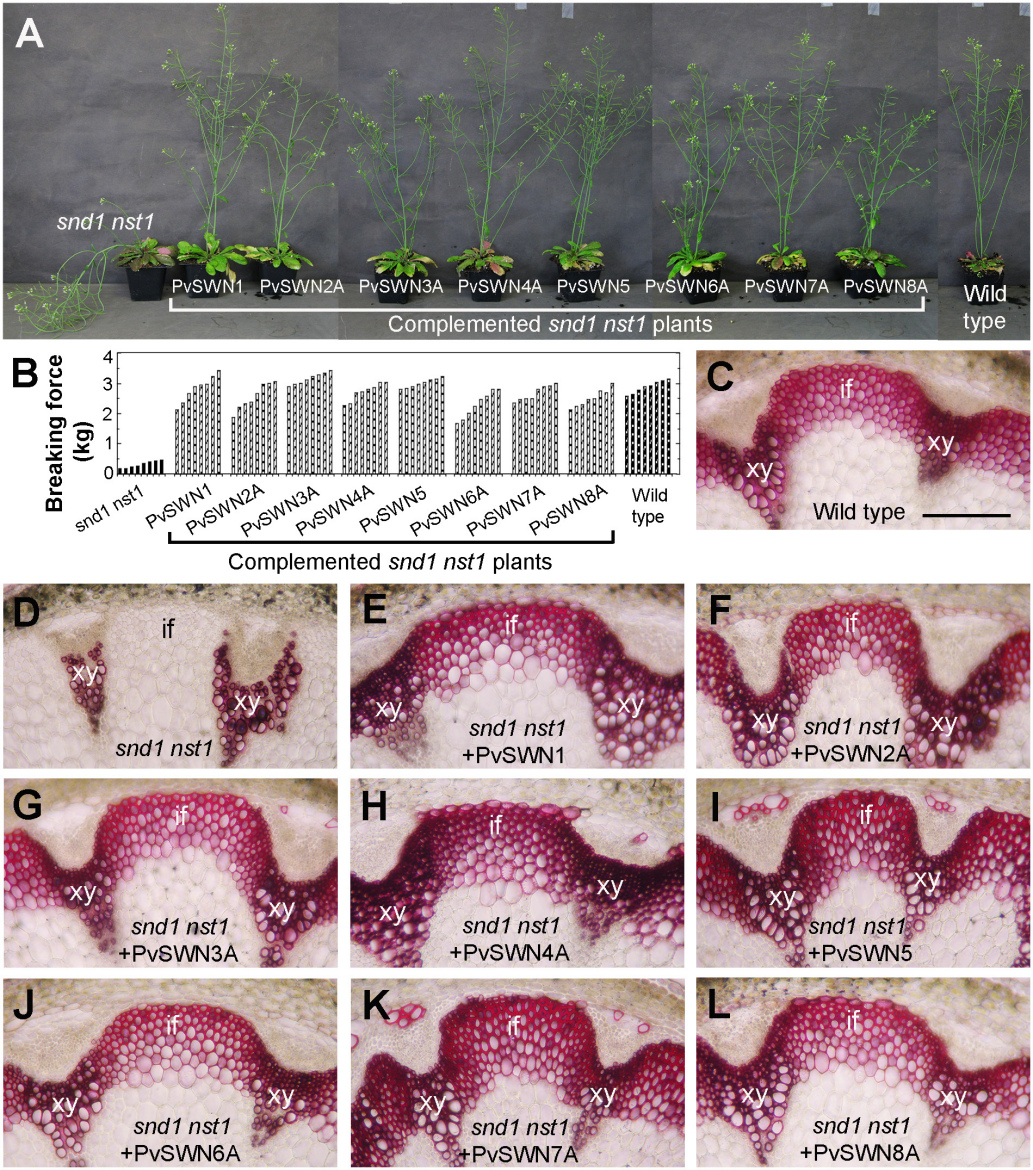
PvSWNs functionally complement the secondary wall defects in the Arabidopsis *snd1 nst1* double mutant. Expression of PvSWNs in the Arabidopsis *snd1 nst1* double mutant was driven by the *SND1* promoter. (A) PvSWNs rescued the pendent inflorescence stems of *snd1 nst1* to the upright form. (B) Measurement of stem breaking strength showing that PvSWNs restored the stem strength of *snd1 nst1* to the wild-type level. Each bar denotes the breaking force of inflorescence stems of individual plants. (C) Cross section of a wild-type stem showing lignified secondary walls in the xylem and interfascicular fibers. (D) Cross section of an *snd1 nst1* stem showing lignified secondary walls only in xylem vessels but not in interfascicular fibers and xylary fibers. (E to L) Cross sections of stems of PvSWN-complemented *snd1 nst1* showing that PvSWNs restored the lignified secondary walls in interfasicular fibers and xylary fibers in *snd1 nst1*. The bottom parts of 7-week-old stems were sectioned and stained for lignin with phloroglucinol-HCl. if, interfascicular fiber; xy, xylem. Bar in (C) = 180 μm for (C to L).

To test whether PvSWNs were capable of complementing the secondary wall defects conferred by the *snd1* and *nst1* mutations, we expressed PvSWNs under the *SND1* promoter in the Arabidopsis *snd1 nst1* double mutant. The *snd1 nst1* double mutant had a pendent stem phenotype due to weakened stem strength caused by lack of lignified secondary walls in interfascicular fibers and xylary fibers [11,13,14] (Fig 8A–8D). Expression of PvSWNs in the *snd1 nst1* double mutant rescued the pendent stem phenotype (Fig 8A) and restored the mechanical strength of stems (Fig 8B). Examination of cross sections of stems showed that PvSWNs completely restored the lignified secondary walls in interfascicular fibers and xylary fibers (Fig 8E–8L). These results provide additional lines of evidence demonstrating that PvSWNs are functional orthologs of Arabidopsis SWNs and are involved in regulating secondary wall biosynthesis.

**Fig 9:**
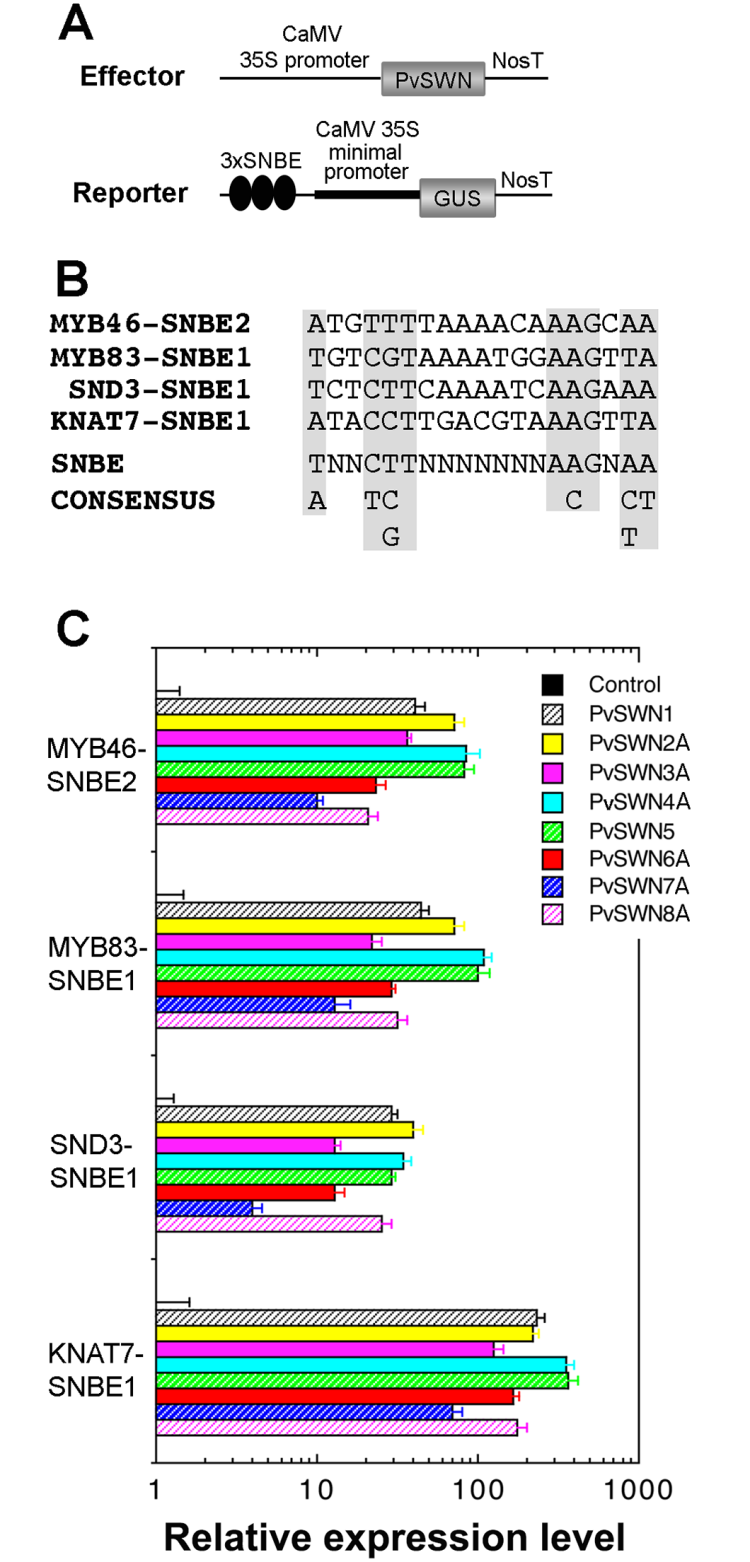
PvSWNs activate the expression of SNBE-driven GUS reporter gene. (A) Diagrams of the effector constructs and the GUS reporter constructs. Three copies of SNBE sequences were ligated upstream of the CaMV 35S minimal promoter linked with the GUS reporter gene. (B) SNBE sequences used for transactivation analysis are from the promoters of *MYB46*, *MYB83*, *SND3* and *KNAT7*. The consensus SNBE sequence is shown at the bottom. (C) Transactivation analysis showing the activation of SNBE-driven GUS reporter gene by PvSWNs. Arabidopsis leaf protoplasts were co-transfected with the reporter and effector constructs as shown in (A) and assayed for GUS activity. The GUS activity in the control protoplasts transfected with the GUS reporter constructs and an empty effector construct without the *PvSWN* sequences is set to 1. Error bars denote the SE of three biological replicates.

It has been shown that Arabidopsis SWNs bind to and activate the 19-bp semi-palindromic Secondary Wall NAC Binding Element (SNBE) sequence in the promoters of their target genes [20]. Using transactivation analysis, we next tested whether PvSWNs were capable of activating the SNBE-driven GUS reporter gene expression. Coexpression of the CaMV 35S promoter-driven PvSWNs and the SNBE-driven GUS reporter gene in Arabidopsis protoplasts demonstrated that PvSWNs effectively activated the GUS reporter gene driven by SNBE sequences from the promoters of *MYB46*, *MYB83*, *SND3* and *KNAT7* (Fig 9), indicating that like Arabidopsis SWNs, PvSWNs bind to the SNBE sites to activate their target genes.

### PvMYB46A is a functional ortholog of Arabidopsis MYB46/MYB83

**Fig 10:**
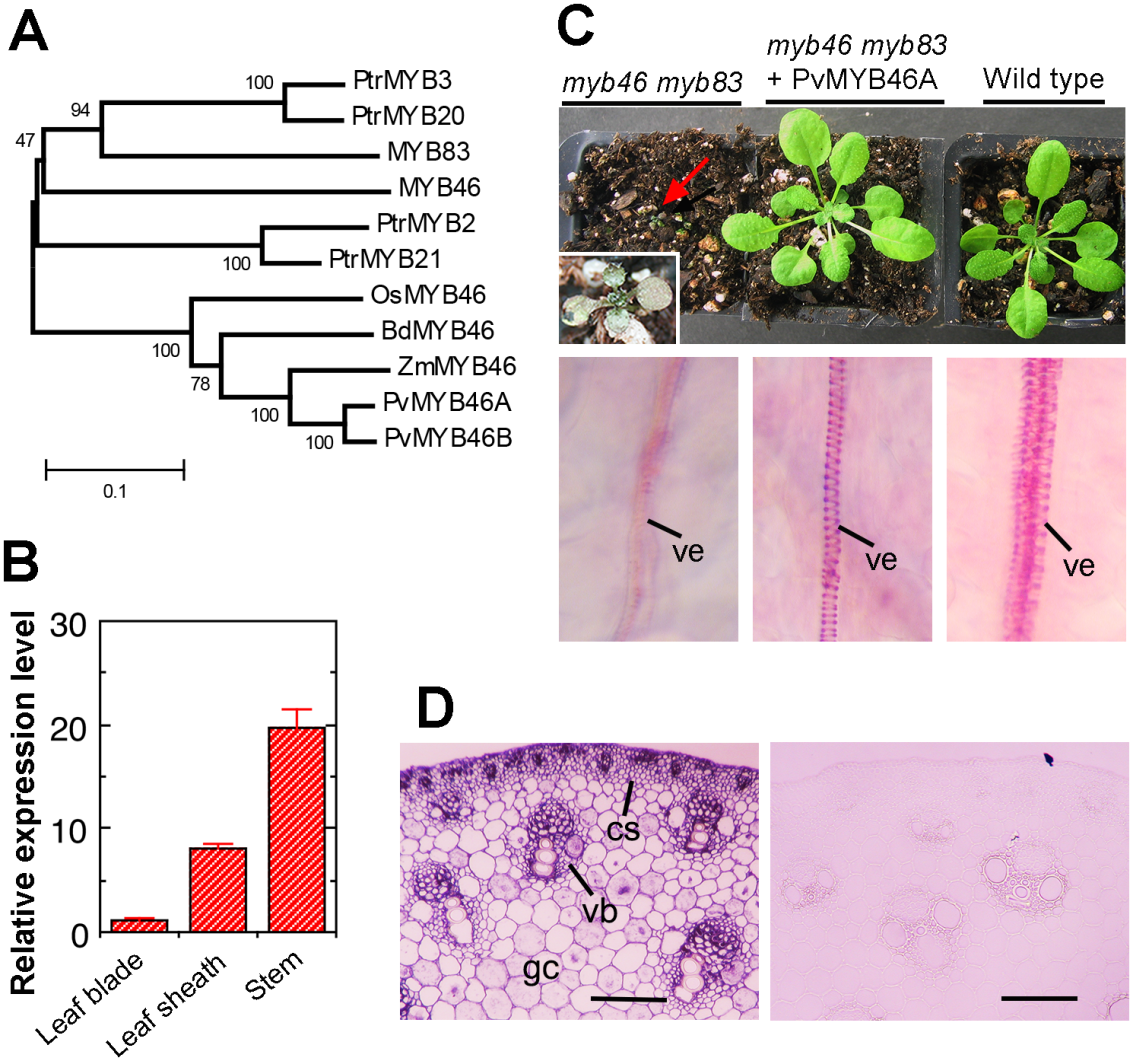
Phylogenetic, expression and functional analyses of *PvMYB46A*. (A) Phylogenetic relationship of PvMYB46A and PvMYB46B with their orthologs from Arabidopsis (MYB46 and MYB83), rice (OsMYB46), maize (ZmMYB46), *Brachypodium* (BdMYB46) and poplar (PtrMYB2, PtrMYB3, PtrMYB20 and PtrMYB21). The phylogenetic tree was constructed with the neighbor-joining algorithm and the bootstrap values resulted from 1,000 replicates are shown as percentages at the nodes. The 0.1 scale denotes 10% change. (B) Quantitative PCR analysis of the expression of *PvMYB46A* in switchgrass organs, including leaf blades, leaf sheaths and stems. The expression level of *PvMYB46A* in leaf blades was set to 1. Error bars denote the SE of three biological replicates. (C) Complementation of the Arabidopsis *myb46 myb83* double mutant by PvMYB46A. The top panel shows the seedling morphology of the severely growth-arrested *myb46 myb83* (arrow; inset showing high magnification of the plant), PvMYB46A-complemented *myb46 myb83*, and the wild type. The bottom panel shows the secondary wall thickening in the leaf veins of the corresponding plants. ve, vessel. (D) In situ localization of *PvMYB46A* mRNA in switchgrass stems. Cross sections of stems were hybridized with digoxigenin-labeled antisense (left panel) or sense (right panel) *PvMYB46A* RNA probes and the hybridization signals, which were detected by alkaline phosphatase-conjugated antibodies, were shown in purple. Note that the majority of cells showing the hybridization signals were matched with those having lignified secondary walls as seen in Fig 1C although those patches of non-lignified cortical cells below the epidermis also showed the hybridization signals, which could be due to non-specific hybridization. cs, cortical sclerenchyma; gc, ground cell; vb, vascular bundle. Bars = 110 μm.

To further expand our understanding of the transcription regulation of secondary wall biosynthesis in switchgrass, we blast-searched the switchgrass genome for MYB sequences that were close homologs of Arabidopsis MYB46/MYB83, which have been shown to be second-level master switches regulating secondary wall biosynthesis [21,23]. It was found that the switchgrass genome harbors two close homologs of MYB46/MYB83, which was named PvMYB46A and PvMYB46B (Fig 10A). They share 89% identity and 91% similarity and like paired PvSWNs, they are likely originated from the increased ploidy level. We thus chose one of them, PvMYB46A, for functional characterization. PvMYB46A/B are more closely grouped together with three other MYB46 homologs from grass species (OsMYB46, BdMYB46 and ZmMYB46) [27,28]; Arabidopsis MYB46/83 are more closely grouped together with four wood-associated MYB46 homologs (PtrMYB3/20/2/21) from poplar [31, 32] (Fig 10A). Quantitative PCR analysis showed that *PvMYB46A* was highly expressed in leaf sheaths and stems (Fig 10B), and *in situ* mRNA localization revealed that in switchgrass stems, *PvMYB46A* was expressed in xylem bundles, cortical sclerenchyma cells and ground cells (Fig 10D), the tissues that underwent lignification (Fig 1C). However, hybridization signals were also seen in those patches of cortical cells below epidermis that did not deposit lignified secondary walls, which could be resulted from non-specific hybridization. In addition, expression of PvMYB46A in the Arabidopsis *myb46 myb83* double mutant completely rescued the mutant phenotypes (Fig 10C), including the arrested seedling growth and the loss of helical secondary wall thickening in leaf veins [23]. These results indicate that PvMYB46A is a functional ortholog of Arabidopsis MYB46/MYB83 involved in regulating secondary wall biosynthesis.

### PvMYB46A overexpression leads to ectopic deposition of secondary walls

**Fig 11:**
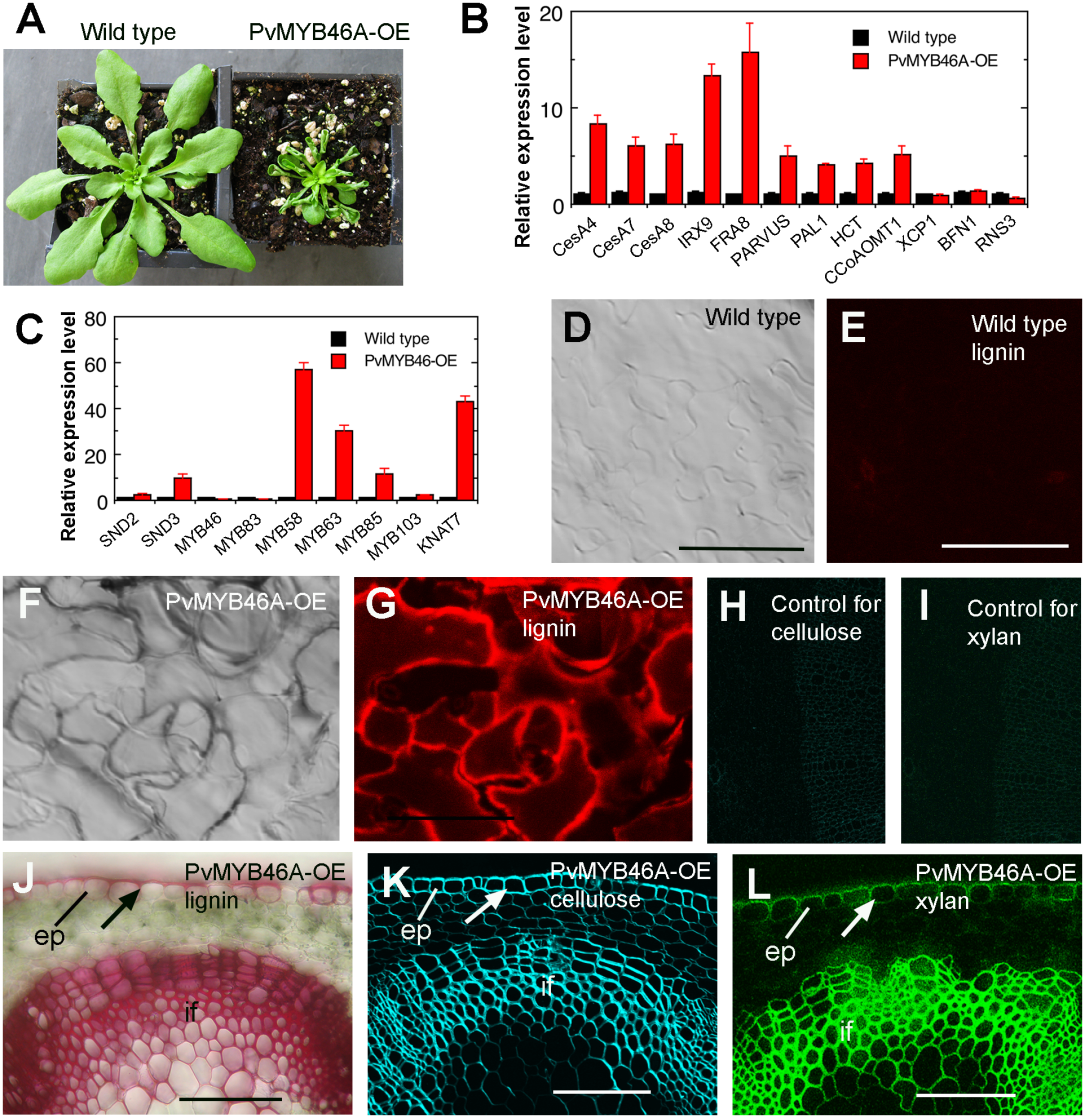
PvMYB46A overexpression induces ectopic deposition of secondary walls in the epidermal cells of leaves and stems of Arabidopsis. (A) Three-week-old seedlings showing the smaller rosette size and curly leaves of the PvMYB46A overexpressor (PvMYB46A-OE) compared with the wild type. (B) Quantitative PCR analysis showing the induction in the expression of secondary wall biosynthetic genes (*CesA4*, *CesA7*, *CesA8*, *FRA8*, *IRX9*, *PARVUS*, *PAL1*, *HCT* and *CCoAOMT1*) in the PvMYB46A overexpressors. No induction of the programmed cell death genes (*XCP1*, *BFN1* and *RNS3*) was detected. The expression level of each gene in the wild type is set to 1 and the error bars represent the SE of three biological replicates. (C) Quantitative PCR analysis showing the induction in the expression of several secondary wall-associated transcription factors in the PvMYB46A overexpressos. (D, E) Differential interference contrast (DIC) image (D) and lignin autofluorescence image (E) of a wild-type leaf showing the absence of lignin signals in the epidermis. (F, G) DIC (F) and lignin autofluorescence image (G) of a PvMYB46A-OE leaf showing lignified walls in the epidermal cells. (H) Control wild-type stem section that was omitted with Calcofluor White showed no fluorescence signals. (I) Control wild-type stem section that was incubated with fluorescein isothiocyanate-conjugated goat-anti-rat secondary antibodies but without LM10 xylan antibody showed no fluorescence signals. (J to L) Ectopic deposition of lignin (J), cellulose (K) and xylan (L) in the epidermal cells of PvMYB46A-OE stems. Cross sections of the stems were stained for lignin with phloroglucinol-HCl, cellulose with Calcofluor White, and xylan with the xylan-specific monoclonal antibody LM10. ep, epidermis; if, interfascicular fiber. Bars = 63 μm in (D) to (G) and 114 μm in (H) to (J).

We next investigated whether PvMYB46A could activate the secondary wall biosynthetic program when overexpressed in Arabidopsis. Transgenic seedlings overexpressing PvMYB46A had small, upward-curling leaves compared with the wild type (Fig 11A), a phenotype similar to that of PvSWN overexpressors (Fig 2A). Quantitative PCR analysis revealed that PvMYB46A overexpression induced the biosynthetic genes for secondary wall cellulose (*CesA4*, *CesA7* and *CesA8*), xylan (*IRX9* and *FRA8*), and lignin (*HCT* and *CCoAOMT*), but not the programmed cell death genes (*BFN1*, *RNS3* and *XCP1*) (Fig 11B). The expression of a number of secondary wall-associated transcription factors, including *SND3*, *MYB58*, *MYB63*, *MYB85* and *KNAT7* was also induced by PvMYB46A (Fig 11C).

Consistent with the induction in the expression of secondary wall biosynthetic genes, PvMYB46A overexpressors had ectopic deposition of lignified secondary walls in the epidermal cells of leaves (Fig 11F and 11G) compared with the epidermis of wild-type leaves, which only displayed weak lignin autofluorescence in guard cells (Fig 11D and 11E). The epidermal cells of stems of PvMYB46A overexpressors also exhibited ectopic deposition of secondary walls with intense staining for lignin, xylan and cellulose (Fig 11H and 11J) compared with the wild type (Figs 4A, 5A and 5J). The control wild-type stem sections omitted with Calcofluor White (Fig 11H) or LM10 xylan antibody (Fig 11I) did not show any fluorescence signals under the same detection conditions. These results indicate that similar to Arabidopsis MYB46/MYB83, PvMYB46A is also able to activate the secondary wall biosynthetic program.

### PvMYB46A activates the SMRE-driven GUS reporter gene

**Fig 12:**
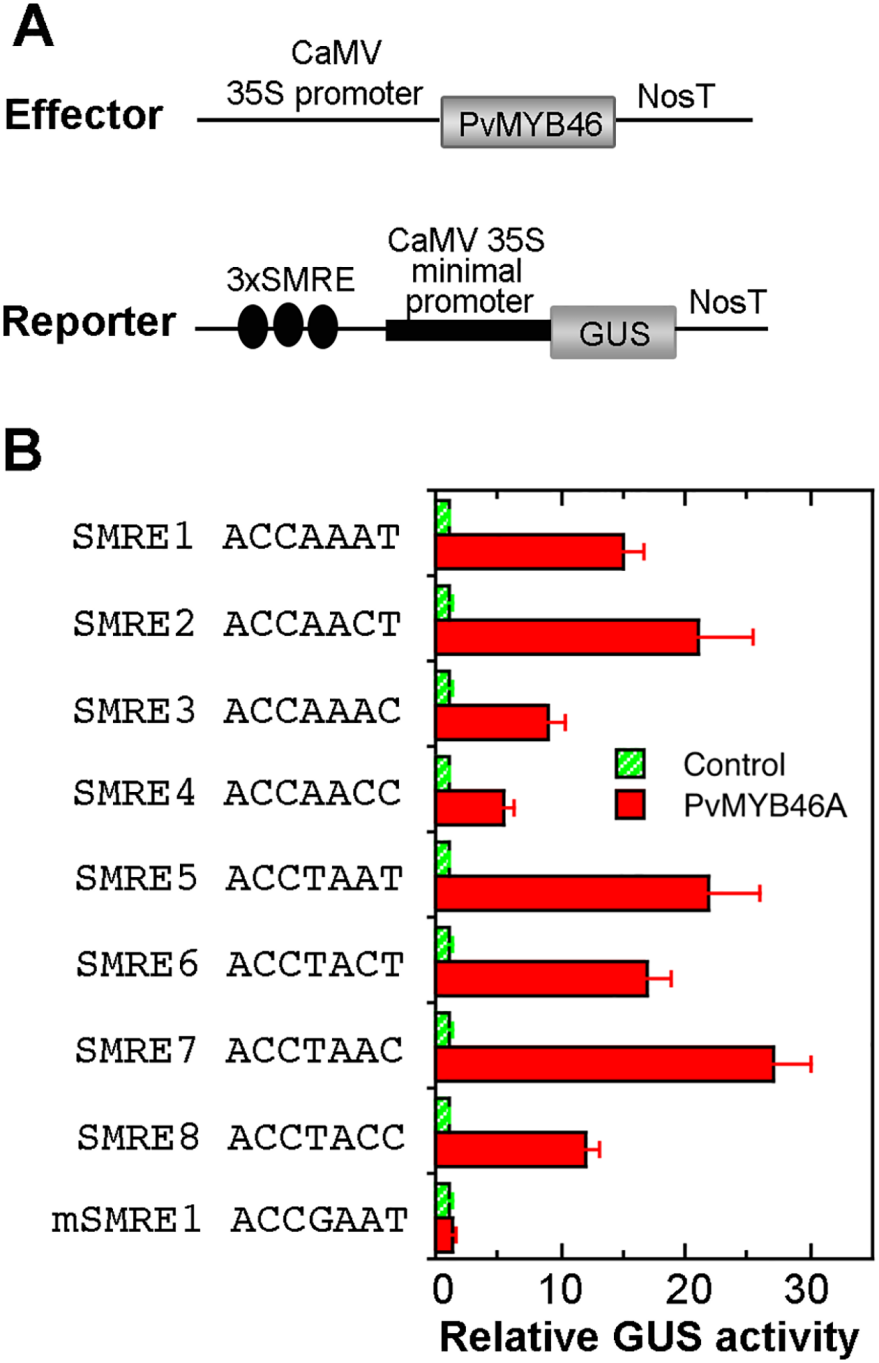
PvMYB46A activates the expression of SMRE-driven GUS reporter gene. (A) Diagrams of the effector construct and the GUS reporter constructs. Three copies of SMRE sequences were ligated upstream of the CaMV 35S minimal promoter linked with the GUS reporter gene. (B) Transactivation analysis showing the activation of SMRE-driven GUS reporter gene by PvMYB46A. The SMRE sequences used in the GUS reporter constructs are shown at the left of the corresponding bars. The effector construct and the GUS reporter constructs as shown in (A) were co-transfected into Arabidopsis leaf protoplasts, which were subsequently assayed for GUS activity. The GUS activity in the control protoplasts transfected with the GUS reporter constructs and an empty effector construct without the *PvMYB46A* sequence is set to 1. Error bars denote the SE of three biological replicates.

It has been shown that Arabidopsis MYB46/MYB83 activate their target genes by binding to the SMRE sequences in their promoters [25]. Transactivation analysis of the SMRE-driven GUS reporter gene revealed that PvMYB46A was able to activate the GUS reporter gene driven by all 8 variants of SMRE sequences (Fig 12A and 12B). The activation levels of the GUS reporter gene varied among the SMRE sequences, indicating that PvMYB46A exhibits differential activation strength toward the 8 variants of SMRE sequences. In contrast, the GUS reporter gene driven by a mutant form of SMRE1 (mSMRE1) showed no activation by PvMYB46A (Fig 12B). Together, these results indicate that like Arabidopsis MYB46/MYB83, PvMYB46A regulates its target gene expression by activating the SMRE sites.

## Discussion

The main above-ground biomass of switchgrass targeted for biofuel production is from stems and leaves. Large amount of lignified secondary walls are deposited in the stems of switchgrass at maturity. In particular, the bundle sheath fibers and cortical sclerenchyma cells are heavily thickened with secondary walls, leaving extremely small intracellular spaces (Fig 1C). To accommodate the heavy deposition of secondary walls in these cells, it is conceivable that switchgrass possesses some mechanisms to sustain the robust secondary wall biosynthetic activity in these cells. One way to achieve this is through a strong, sustained activation of the expression of secondary wall biosynthetic genes, which can be fulfilled by transcription factors activating their expression. Our functional studies on switchgrass NAC and MYB transcription factors have revealed that among the secondary-wall NAC and MYB master switches, three of them, namely PvSWN4A, PvSWN5 and PvSWN7A, exhibit a strong activation of the secondary wall biosynthetic program (Figs 3–5), and when overexpressed in Arabidopsis, they could lead to ectopic deposition of extremely thick secondary walls that are up to three times thicker than the walls of wild-type fiber cells (Fig 3). This finding indicates that PvSWN4A/5/7A are strong master switches activating secondary wall biosynthesis leading to massive deposition of secondary walls. If so, these PvSWNs could potentially be used as molecular tools to induce massive deposition of secondary walls in switchgrass and other biofuel crops so that the lignocellulosic biomass yield per land acreage could be increased.

The Arabidopsis SWNs can be divided into two groups based on their expression patterns, i.e., vessel-specific SWNs (VNDs) and fiber-specific SWNs (SND1/NSTs). Orthologs of both vessel-specific VNDs and fiber-specific SND1/NSTs are present in switchgrass and other grass species including rice, maize and *Brachypodium* (Fig 1A) [27–29]. However, SWNs from switchgrass, rice and maize appear to be expressed in all secondary wall-forming cells including both vessels and fibers in stems [27,29], indicating that unlike those in Arabidopsis, SWNs in grass species did not diverge into vessel- or fiber-specific groups.

The Arabidopsis vessel-specific SWNs (VNDs) have been shown to induce the expression of not only secondary wall biosynthetic genes, but also programmed cell death genes [15,17,20]. When overexpressed in Arabidopsis, PvSWN3A to PvSWN8A, which are orthologs of VNDs, were also capable of activating programmed cell death genes. In contrast, PvSWN1 and PvSWN2A, which are orthologs of Arabidopsis fiber-specific SND1 and NST1/2, had little effect on the expression of programmed cell death genes. Activation of programmed cell death genes by *Brachypodium* orthologs of VNDs but not those of SND1/NST1/2 was also observed [28]. These findings indicate that although switchgrass orthologs of the Arabidopsis VNDs do not exhibit vessel-specific expression [27,29], they retained similar functionality as VNDs in activating not only secondary wall biosynthetic genes but also programmed cell death genes.

Our finding that PvMYB64A is sufficient to activate secondary wall biosynthetic pathways for cellulose, xylan and lignin provides further molecular evidence demonstrating the evolutionary conservation of the SWNs- and MYB46/83-mediated transcriptional network. So far, several MYB46 homologs from dicots (poplar, *Eucalyptus* and pine) [16,31–35] and grass species (rice and maize) [27] have been shown to be functional orthologs of MYB46/83 regulating secondary wall biosynthesis. All these MYB46 homologs activate their target genes by binding to the SMRE sites, indicating that the functional conservation is achieved through their binding to the common cis-elements for transcriptional activation. It is interesting to note that PtMYB46A activates the expression of secondary wall biosynthetic genes but not the genes involved in programmed cell death. This finding implies that although PvSWNs and PtMYB46 co-regulate the secondary wall biosynthetic program, PtMYB46 is unlikely involved in activating the programmed cell death program during vessel development, which is consistent with the previous finding showing that in Arabidopsis, the vessel-specific VNDs directly regulate the expression of programmed cell death genes through binding and activating the SNBE sites [15,17,18,20].

Considerable interest has recently been focused on developing perennial grasses, such as switchgras and *Miscanthus*, as a source of renewable energy; therefore, a better understanding of genes involved in the biosynthesis of grass cell walls is deemed necessary [36]. Although secondary cell walls from both grasses and dicots are made of cellulose, hemicelluloses and lignin, they differ in their composition [37]. For example, xylan from grasses is substituted with arabinosyl residues, which are often cross-linked with lignin via ferulic acid, whereas xylan from dicots has few arabinosyl substitutions. In addition, lignin from grasses contain a much higher amount of *p*-hydroxylphenyl units than that from dicots [37]. The heterogeneity of cell walls can be controlled by cell wall biosynthetic genes as well as transcriptional regulators of the biosynthetic genes. So far, only a few studies have been devoted to the transcriptional regulation of grass cell wall biosynthesis [27–29, 38,39]. Available evidence indicates that the NAC and MYB master transcriptional switches activating the secondary wall biosynthetic program are conserved between grasses and dicots [27–29]. Furthermore, it appears that the grass orthologs of the Arabidopsis MYB transcriptional repressors for lignin biosynthesis are also functionally conserved [6,40,41]. However, since grass cell walls differ from those of dicots in their composition, it is conceivable that the transcription regulation of secondary wall biosynthesis in grass species might have some divergence from that in dicots. Further identification and functional characterization of switchgrass regulatory genes controlling secondary wall biosynthesis will not only help decipher how grass cell walls are constructed but also offer additional molecular tools to genetically modify grass cell wall composition better suited for biofuel production.

## Methods

### Gene expression analysis

Total RNA was isolated from switchgrass and Arabidopsis tissues with a Qiagen RNA isolation kit (Qiagen). After treatment with DNase I, the total RNA was converted into first strand cDNAs, which were then used as templates for PCR analysis. The real-time quantitative PCR was performed with the QuantiTect SYBR Green PCR kit (Clontech) using first strand cDNAs as templates. The PCR primers for *PvSWN1* are 5’-ggccgcgtcgtacgagctcaa-3’ and 5’-ctacacgttgttcatcaagtccgc-3’, those for *PvSWN2A* are 5’-gatcacagactgggccatgatgga-3’ and 5’-ctacagcgacacgtggctcag-3’, those for *PvSWN3A* are 5’-tccaagctgcaggtgcatcatctc-3’ and 5’-tcacatgacaaggtcattgctgtt-3’, those for *PvSWN4A* are 5’-gcagcaggagtactattgcggcaa-3’ and 5’-tgctgccacctatggttcctg-3’, those for *PvSWN5* are 5’-acaagttcgtcgcgtcgcagctca-3’ and 5’-tcatttctcgaacacgcagagtcc-3’, those for *PvSWN6A* are 5’-ccacactccaaccatgcagcctca-3’ and 5’-ctgctatttccacaagtcgttatc-3’, those for *PvSWN7A* are 5’-tcagctcatggatgatgcagtcga-3’ and 5’-tcatcacttccatggatcaacttg-3’, those for *PvSWN8A* are 5’-cggcgatggaggctgcgtacatga-3’ and 5’-ctatttccacaggtcagcctcgct-3’, and those for *PvMYB46A* are 5’-aacaatgagagcaacatcacagac-3’ and 5’-tcattcaacttggaaatcaaggaa-3’. The PCR primers for Arabidopsis secondary wall-associated transcription factors and secondary wall biosynthetic genes were according to Zhong et al. (2006). The relative expression level of each gene was calculated by normalizing the PCR threshold cycle number of each gene with that of an actin reference gene from switchgrass or the *EF1α* reference gene from Arabidopsis. The data were the average of three biological replicates.

### In situ hybridization

Elongating switchgrass stems were fixed in 2.5% formaldehyde and 0.5% glutaraldehyde, embedded in paraffin, and sectioned (12 μm thick) according to McAbee et al. [42] and Zhou et al. [43]. The 250-bp 3’ untranslated sequences of PvSWN cDNAs were used for synthesis of digoxigenin-labeled antisense and sense RNA probes with the DIG RNA labeling mix (Roche). The primers used for PCR-amplification of the 250-bp 3’ untranslated sequences are 5’- cacctcaacgggcaggcggccga-3’ and 5’-ggccggacggctacagcgacacgt-3’ for PvSWN2A, 5’- tgatgcaattaatcaaacataatacct-3’ and 5’-tgattcatgaagcatctgagttct-3’ for PvSWN7A, 5’- ctgctgaagaacgtacacgaaccca-3’ and 5’-tacttgtttattgtgcataagtat-3’ for PvSWN8A, and 5’- ttgggagggcccaactccagctggct-3’ and 5’-agctcaagatttgatttaattatt-3’ for PvMYB46A. Stem sections were hybridized with the antisense and sense probes and the hybridization signals were detected by incubation with alkaline phosphatase-conjugated antibodies against digoxygenin and subsequent color development with alkaline phosphatase substrates.

### Overexpression of PvSWNs and PvMYB46A

The full-length cDNAs of *PvSWNs* and *PvMYB46A* were ligated downstream of the CaMV 35S promoter in pBI121 to create overexpression constructs. The constructs were introduced into wild-type *Arabidopsis thaliana* (ecotype Columbia) by agrobacterium-mediated transformation. For each construct, at least 30 transgenic Arabidopsis plants were generated and used for morphological and histological analyses.

### Complementation of Arabidopsis mutants

The full-length cDNAs of *PvSWNs* driven by the 3-kb *SND1* promoter were cloned into the pGPTV-HPT vector and introduced into the Arabidopsis *snd1 nst1* double mutant [14]. For each construct, at least 30 transgenic plants were generated and 8 of them were examined for stem strength and restoration of secondary walls in fibers. For stem strength analysis, the basal parts of the main inflorescence of 7-week-old plants were measured for breaking force using a digital force/length tester [44]. The breaking force was calculated as the force needed to break apart a stem segment.

The full-length cDNA of *PvMYB46A* driven by the 3-kb *MYB46* promoter was cloned into the pGPTV-HPT vector and introduced into the *myb46* (-/-; homozygous) *myb83* (+/-; heterozygous) double mutant [23]. Transgenic plants were screened for double homozygous *myb46 (-/-) myb83 (-/-)* mutants by PCR-based genotyping as described previously [23]. At least 10 transgenic plants with the *myb46 (-/-) myb83 (-/-)* background were examined for plant growth and vessel morphology phenotypes.

### Histology

Tissues were fixed in 2% formaldehyde and embedded in Low Viscosity (Spurr's) resin (Electron Microscopy Sciences) and cut into thin sections with a microtome as described [45]. One-μm-thick sections were stained with toluidine blue for examination of cell wall morphology using light microscopy. Visualization of lignin in the stems were done by staining 50-μm-thick sections with phloroglucinol-HCl, which stains lignin bright red. For lignin autofluorescence visualization, leaves were cleared in methanol and examined using a UV fluorescence microscope [13]. Visualization of secondary wall cellulose was achieved by incubating 1-μm-thick sections with 0.01% Calcofluor White [46]. The fluorescence signals for cellulose staining were detected with excitation at 360 nm and emission at 480 nm under a confocal microscope. No bright white signals were observed in sections without Calcofluor White staining under the same detection conditions. For immunostaining of xylan, 1-μm-thick sections were first incubated with the monoclonal LM10 antibody and then with fluorescein isothiocyanate-conjugated goat-anti-rat secondary antibodies according to McCartney et al. [47]. The fluorescence signals for xylan immunostaining were detected with excitation at 488 nm and emission at 600 nm under a confocal microscope. The control sections were omitted with the LM10 antibody and no fluorescence signals were observed under the same detection conditions.

### Transactivation analysis

The reporter and the effector constructs were co-transfected into Arabidopsis leaf protoplasts [48]. A construct containing the firefly luciferase gene driven by the CaMV 35S promoter was included in each transfection for determination of the transfection efficiency. After 20-hr incubation, protoplasts were lysed and the supernatants were assayed for GUS and luciferase activities. The GUS activity was normalized against the luciferase activity in each transfection, and the data are the average of three biological replicates.

### Statistical analysis

The experimental data of the quantitative PCR analysis and GUS activity assay were subjected to statistical analysis using the Student’s *t* test program (http://www.graphpad.com/quickcalcs/ttest1.cfm), and the quantitative difference between the two groups of data for comparison in each experiment was found to be statistically significant (p < 0.001).

### Accession numbers

The gene identification numbers (*Panicum virgatum v1*.*1*) and the GenBank accession numbers for the genes used in this study are PvSWN1 (Pavir.J07835; KT075080), PvSWN2A (Pavir.J20698; KT075081), PvSWN2B (Pavir.J21162; KT075082), PvSWN3A (Pavir.J20890; KT075083), PvSWN3B (Pavir.J31179; KT075084), PvSWN4A (Pavir.Ib02477; KT075085), PvSWN4B (Pavir.Ia02924; KT075086), PvSWN5 (Pavir.Ib00161; KT075087), PvSWN6A (Pavir.J07126; KT075088), PvSWN6B (Pavir.Gb00744; KT075089), PvSWN7A (Pavir.J26987; KT075090), PvSWN7B (Pavir.Da02426; KT075091), PvSWN8A (Pavir.J09314; KT075092), PvSWN8B (Pavir.J39804; KT075093), PvMYB46A (Pavir.J11191; KT075094), and PvMYB46B (Pavir.Ca02370; KT075095). PvSWN1, PvSWN2A, PvSWN2B and PvSWN5 were previously described as PvNAC055, PvNAC101, PvNAC61/62 and PvNAC068, respectively [49]. PvMYB46A and PvMYB46B were previously described as AP13ISTG55477 and AP13ISTG55479, respectively [50].

## References

[1] McLaughlin SB, Kszos LA (2005) Development of switchgrass (*Panicum virgatum*) as a bioenergy feedstock in the United States. Biomass Bioenergy 28: 515–535.

[2] Parrish D, Fike JH (2005) The biology and agronomy of switchgrass for biofuels. Critic Rev Plant Sci 24: 423–459.

[3] Fu C, Mielenz JR, Xiao X, Ge Y, Hamilton CY, Rodriguez M, et al 2011 Genetic manipulation of lignin reduces recalcitrance and improves ethanol production from switchgrass. Proc Natl Acad Sci USA 108: 3803–3808. 10.1073/pnas.1100310108 21321194 PMC3048149

[4] Xu B, Escamilla-Trevino LL, Sathitsuksanoh N, Shen Z, Shen H, Zhang YH, et al (2011) Silencing of 4-coumarate:coenzyme A ligase in switchgrass leads to reduced lignin content and improved fermentable sugar yields for biofuel production. New Phytol 192: 611–625. 10.1111/j.1469-8137.2011.03830.x 21790609

[5] Saathoff AJ, Sarath G, Chow EK, Dien BS, Tobias CM (2011) Downregulation of cinnamyl-alcohol dehydrogenase in switchgrass by RNA silencing results in enhanced glucose release after cellulase treatment. PloS ONE 6: e16416 10.1371/journal.pone.0016416 21298014 PMC3029337

[6] Shen H, He X, Poovaiah CR, Wuddineh WA, Ma J, Mann DG, et al (2012) Functional characterization of the switchgrass (*Panicum virgatum*) R2R3-MYB transcription factor PvMYB4 for improvement of lignocellulosic feedstocks. New Phytol 193: 121–136. 10.1111/j.1469-8137.2011.03922.x 21988539

[7] Shen H, Mazarei M, Hisano H, Escamilla-Trevino L, Fu C, Pu Y, et al (2013) A genomics approach to deciphering lignin biosynthesis in switchgrass. Plant Cell 25: 4342–4361. 10.1105/tpc.113.118828 24285795 PMC3875722

[8] Poovaiah CR, Mazarei M, Decker SR, Turner GB, Sykes RW, Davis MF, et al (2015) Transgenic switchgrass (*Panicum virgatum* L.) biomass is increased by overexpression of switchgrass sucrose synthase (PvSUS1). Biotechnol J 10: 552–563. 10.1002/biot.201400499 25327983

[9] Zhong R, Ye Z-H (2014) Complexity of the transcriptional network controlling secondary wall biosynthesis. Plant Science 229: 193–207. 10.1016/j.plantsci.2014.09.009 25443846

[10] Kubo M, Udagawa M, Nishikubo N, Horiguchi G, Yamaguchi M, Ito J, et al (2005) Transcription switches for protoxylem and metaxylem vessel formation. Genes Dev 19: 1855–1860. 16103214 10.1101/gad.1331305 PMC1186185

[11] Mitsuda N, Iwase A, Yamamoto H, Yoshida M, Seki M, Shinozaki K, et al (2007) NAC transcription factors, NST1 and NST3, are key regulators of the formation of secondary walls in woody tissues of Arabidopsis. Plant Cell 19: 270–280. 17237351 10.1105/tpc.106.047043 PMC1820955

[12] Mitsuda N, Seki M, Shinozaki K, Ohme-Takagi M (2005) The NAC transcription factors NST1 and NST2 of Arabidopsis regulates secondary wall thickening and are required for anther dehiscence. Plant Cell 17: 2993–3006. 16214898 10.1105/tpc.105.036004 PMC1276025

[13] Zhong R, Demura T, Ye Z-H (2006) SND1, a NAC domain transcription factor, is a key regulator of secondary wall synthesis in fibers of Arabidopsis. Plant Cell 18: 3158–3170. 17114348 10.1105/tpc.106.047399 PMC1693950

[14] Zhong R, Richardson EA, Ye Z-H (2007) Two NAC domain transcription factors, SND1 and NST1, function redundantly in regulation of secondary wall synthesis in fibers of Arabidopsis. Planta 225: 1603–1611. 17333250 10.1007/s00425-007-0498-y

[15] Ohashi-Ito K, Oda Y, Fukuda H (2010) Arabidopsis VASCULAR-RELATED NAC-DOMAIN6 directly regulates the genes that govern programmed cell death and secondary wall formation during xylem differentiation. Plant Cell 22: 3461–3473. 10.1105/tpc.110.075036 20952636 PMC2990123

[16] Zhong R, Lee C, Ye Z-H (2010) Evolutionary conservation of the transcriptional network regulating secondary cell wall biosynthesis. Trends Plant Sci 15: 625–631. 10.1016/j.tplants.2010.08.007 20833576

[17] Yamaguchi M, Mitsuda N, Ohtani M, Ohme-Takagi M, Kato K, Demura T (2011) VASCULAR-RELATED NAC-DOMAIN7 directly regulates the expression of a broad range of genes for xylem vessel formation. Plant J 66: 579–590. 10.1111/j.1365-313X.2011.04514.x 21284754

[18] Zhou J, Zhong R, Ye Z-H (2014) Arabidopsis NAC domain proteins, VND1 to VND5, are transcriptional regulators of secondary wall biosynthesis in vessels. PLoS One 9: e105726 10.1371/journal.pone.0105726 25148240 PMC4141820

[19] Zhong R, Ye Z-H (2015) The Arabidopsis NAC transcription factor NST2 functions together with SND1 and NST1 to regulate secondary wall biosynthesis in fibers of inflorescence stems. Plant Signal Behav 10: e989746 10.4161/15592324.2014.989746 25751728 PMC4622706

[20] Zhong R, Lee C, Ye Z-H (2010) Global analysis of direct targets of secondary wall NAC master switches in Arabidopsis. Mol Plant 3: 1087–1103. 10.1093/mp/ssq062 20935069

[21] Zhong R, Richardson EA, Ye Z-H (2007) The MYB46 transcription factor is a direct target of SND1 and regulates secondary wall biosynthesis in Arabidopsis. Plant Cell 19: 2776–2792. 17890373 10.1105/tpc.107.053678 PMC2048704

[22] Zhong R, Lee C, Zhou J, McCarthy RL, Ye Z-H (2008) A battery of transcription factors involved in the regulation of secondary cell wall biosynthesis in Arabidopsis. Plant Cell 20: 2763–2782. 10.1105/tpc.108.061325 18952777 PMC2590737

[23] McCarthy RL, Zhong R, Ye Z-H (2009) MYB83 is a direct target of SND1 and acts redundantly with MYB46 in the regulation of secondary cell wall biosynthesis in Arabidopsis. Plant Cell Physiol 50: 1950–1964. 10.1093/pcp/pcp139 19808805

[24] Zhou J, Lee C, Zhong R, Ye Z-H (2009) MYB58 and MYB63 are transcriptional activators of the lignin biosynthetic pathway during secondary cell wall formation in Arabidopsis. Plant Cell 21: 248–266. 10.1105/tpc.108.063321 19122102 PMC2648072

[25] Zhong R, Ye Z-H (2012) MYB46 and MYB83 bind to the SMRE sites and directly activate a suite of transcription factors and secondary wall biosynthetic genes. Plant Cell Physiol 53: 368–380. 10.1093/pcp/pcr185 22197883

[26] Kim WC, Ko JH, Han KH (2012) Identification of a cis-acting regulatory motif recognized by MYB46, a master transcriptional regulator of secondary wall biosynthesis. Plant Mol Biol 78: 489–501. 10.1007/s11103-012-9880-7 22271306

[27] Zhong R, Lee C, McCarthy RL, Reeves CK, Jones EG, Ye Z-H (2011) Transcriptional activation of secondary wall biosynthesis by rice and maize NAC and MYB transcription factors. Plant Cell Physiol 52: 1856–1871. 10.1093/pcp/pcr123 21908441

[28] Valdivia ER, Herrera MT, Gianzo C, Fidalgo J, Revilla G, Zarra I, et al (2013) Regulation of secondary wall synthesis and cell death by NAC transcription factors in the monocot *Brachypodium distachyon* . J Exp Bot 64: 1333–1343. 10.1093/jxb/ers394 23386682 PMC3598421

[29] Yoshida K, Sakamoto S, Kawai T, Kobayashi Y, Sato K, Ichinose Y, et al (2013) Engineering the *Oryza sativa* cell wall with rice NAC transcription factors regulating secondary wall formation. Front Plant Sci 4: 383 10.3389/fpls.2013.00383 24098302 PMC3787547

[30] Zhong R, Ye Z-H (2015) Secondary cell walls: biosynthesis, patterned deposition and transcriptional regulation. Plant Cell Physiol 56: 195–214. 10.1093/pcp/pcu140 25294860

[31] Zhong R, McCarthy RL, Haghighat M, Ye Z-H (2013) The poplar MYB master switches bind to the SMRE site and activate the secondary wall biosynthetic program during wood formation. PLoS ONE 8: e69219 10.1371/journal.pone.0069219 23922694 PMC3726746

[32] McCarthy RL, Zhong R, Fowler S, Lyskowski D, Piyasena H, Carleton K, et al (2010) The poplar MYB transcription factors, PtrMYB3 and PtrMYB20, are involved in the regulation of secondary wall biosynthesis. Plant Cell Physiol 51: 1084–1090. 10.1093/pcp/pcq064 20427511

[33] Patzlaff A, McInnis S, Courtenay A, Surman C, Newman LJ, Smith C, et al (2003) Characterization of a pine MYB that regulates lignification. Plant J 36: 743–754. 14675440 10.1046/j.1365-313x.2003.01916.x

[34] Goicoechea M, Lacombe E, Legay S, Mihaljevic S, Rech P, Jauneau A, et al (2005) EgMYB2, a new transcriptional activator from Eucalyptus xylem, regulates secondary cell wall formation and lignin biosynthesis. Plant J 43: 553–567. 16098109 10.1111/j.1365-313X.2005.02480.x

[35] Bomal C, Bedon F, Caron S, Mansfield SD, Levasseur C, Cooke JK, et al (2008) Involvement of *Pinus taeda* MYB1 and MYB8 in phenylpropanoid metabolism and secondary cell wall biogenesis: a comparative in planta analysis. J Exp Bot 59: 3925–3939. 10.1093/jxb/ern234 18805909 PMC2576632

[36] Nageswara-Rao M, Soneji JR, Kwit C, Stewart CN Jr. (2013) Advances in biotechnology and genomics of switchgrass. Biotechnol Biofuels 6: 77 10.1186/1754-6834-6-77 23663491 PMC3662616

[37] Vogel J (2008) Unique aspects of the grass cell wall. Curr Opin Plant Biol 11: 301–307. 10.1016/j.pbi.2008.03.002 18434239

[38] Bosch M, Mayer CD, Cookson A, Donnison IS (2011) Identification of genes involved in cell wall biogenesis in grasses by differential gene expression profiling of elongating and non-elongating maize internodes. J Exp Bot 62: 3545–3561. 10.1093/jxb/err045 21402660 PMC3130177

[39] Hirano K, Kondo M, Aya K, Miyao A, Sato Y, Antonio BA, et al (2013) Identification of transcription factors involved in rice secondary cell wall formation. Plant Cell Physiol 54: 1791–1802. 10.1093/pcp/pct122 24089432

[40] Fornale S, Shi X, Chai C, Encina A, Irar S, Capellades M, et al (2010) ZmMYB31 directly represses maize lignin genes and redirects the phenylpropanoid metabolic flux. Plant J 64: 633–644. 10.1111/j.1365-313X.2010.04363.x 21070416

[41] Sonbol FM, Fornale S, Capellades M, Encina A, Tourino S, Torres J-L, et al (2009) The maize ZmMYB42 represses the phenylpropanoid pathway and affects the cell wall structure, composition and degradability in *Arabidopsis thaliana* . Plant Mol Biol 70: 283–296. 10.1007/s11103-009-9473-2 19238561

[42] McAbee JM, Kuzoff RK, Gasser CS (2005) Mechanisms of derived unitegmy among Impatiens species. Plant Cell 17: 1674–1684. 15849275 10.1105/tpc.104.029207 PMC1143069

[43] Zhou GK, Zhong R, Richardson EA, Morrison WH 3rd, Nairn CJ, Wood-Jones A, et al (2006) The poplar glycosyltransferase GT47C is functionally conserved with Arabidopsis *Fragile fiber8* . Plant Cell Physiol 47: 1229–1240. 16887843 10.1093/pcp/pcj093

[44] Zhong R, Taylor JJ, Ye Z-H (1997) Disruption of interfascicular fiber differentiation in an Arabidopsis mutant. Plant Cell 9: 2159–2170. 9437861 10.1105/tpc.9.12.2159 PMC157065

[45] Burk DH, Zhong R, Morrison WH III, Ye Z-H (2006) Disruption of cortical microtubules by overexpression of green fluorescent protein-tagged α-tubulin 6 causes a marked reduction in cell wall synthesis. J Integr Plant Biol 48: 85–98.

[46] Hughes J, McCully ME (1975) The use of an optical brightener in the study of plant structure. Stain Technol 50: 319–329. 54956 10.3109/10520297509117082

[47] McCartney L, Marcus SE, Knox JP (2005) Monoclonal antibodies to plant cell wall xylans and arabinoxylans. J Histochem Cytochem 53: 543–546. 15805428 10.1369/jhc.4B6578.2005

[48] Sheen J (2001) Signal transduction in maize and Arabidopsis mesophyll protoplasts. Plant Physiol 127: 1466–1475. 11743090 PMC1540179

[49] Shen H, Yin Y, Chen F, Xu Y, Dixon R (2009) A bioinformatic analysis of NAC genes for plant cell wall development in relation to lignocellulosic bioenergy production. BioEnergy Res 2: 217–232.

[50] Zhao K, Bartley L (2014) Comparative genomic analysis of the R2R3 MYB secondary cell wall regulators of Arabidopsis, poplar, rice, maize, and switchgrass. BMC Plant Biol 14: 135 10.1186/1471-2229-14-135 24885077 PMC4057907

